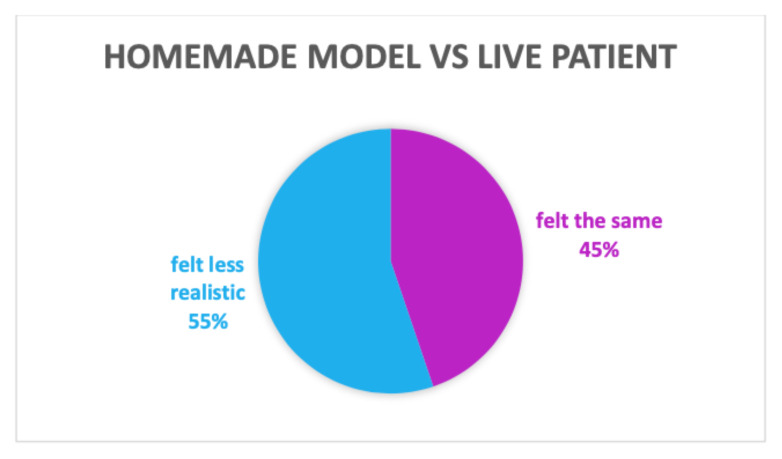# A Homemade, Cost-Effective, Realistic Pelvic Exam Model

**DOI:** 10.21980/J8HM0F

**Published:** 2023-07-31

**Authors:** Jessie Godsey, Ilya Kott, Adrienne Payden, Patricia Ward

**Affiliations:** * Ascension Macomb Oakland, Department of Emergency Medicine, Warren, MI

## Abstract

**Audience:**

This innovation is intended to instruct medical students, residents of all levels, and mid-level practitioners.

**Background:**

Pelvic examinations are essential components to clinical practice but are challenging to teach, learn, and practice on live patients secondary to patient comfort because this is an invasive procedure.^1^ Resident physicians and medical students traditionally learn these methods through observation while actively working in their department or clinics.^2^ Simulation models can improve a provider’s competency and confidence performing pelvic examinations which improve patient comfort and exam accuracy.^3^ One barrier to simulation training is the cost of the pelvic simulator models. A basic pelvic exam simulator costs $365.^4^ The cost is high, therefore limiting the availability of a simulation model accessible to residency programs across the country. This barrier to pelvic models was overcome by developing a homemade alternative for cervical examination and collection of screening swabs. The model created can be easily manufactured by students, residents, and faculty alike for less than $20 and approximately two hours of manufacturing time. A literature review was conducted to find similar products and other production methods for a pelvic examination model. No comparable models were found.

This is a guide to utilizing supplies from a local dollar store combined with home recycling products and a few common crafting tools to create a realistic pelvic examination model.

**Educational Objectives:**

After utilizing this pelvic examination model, the learner will be able to: 1) demonstrate ability to perform a pelvic examination comfortably and safely, 2) demonstrate ability to obtain a cervical swab on female patients, and 3) show proficient understanding of female anatomy.

**Educational Methods:**

The pelvic exam model is utilized to effectively teach proper technique for pelvic examinations. This model can be utilized to teach medical students, incoming residents, and new mid-levels. Senior residents, experienced mid-levels, or attendings who are experienced in completing pelvic examinations can easily utilize this model to teach proper technique.

**Research Methods:**

The data for this study was collected from a single graduate medical education program in Detroit, Michigan. This was designed as a single blind survey where the reviewer’s identities were kept anonymous from the data collectors. Surveys were collected from attendings, residents, mid-level providers, and medical students across specialties of emergency medicine, family medicine, obstetrics and gynecology.

**Results:**

A total of 77 individuals tested the homemade model and compared it to a pelvic exam on a live patient as well as a commercial pelvic exam model. Survey results showed the low-cost homemade model was just as effective as a commercially manufactured model, with some respondents saying the DIY model was more effective and more realistic. Comparing the commercial models to the homemade model, 54 of the 77 participants had experience with a commercial model. In the survey when compared to a commercial model, 57% of the participants felt the examination was the same, and 31% indicated the homemade model felt more realistic.

**Discussion:**

Overall, the homemade cost-effective model is comparable if not more realistic to more expensive commercial models. The main take away of this innovation, to remember it is possible to create cost-effective models for realistic, educational learning. This model has one limitation because it is not suitable for a bimanual examination, but it can be expanded to allow for bimanual examination.

**Topics:**

Pelvic examination, cervical examination, creative simulation models.

## USER GUIDE

[Table t4-jetem-8-3-i1]

**Table t4-jetem-8-3-i1:** 

List of Resources:	
Abstract	1
User Guide	3

**Learner Audience:**

Medical Students, Interns, Junior Residents, Senior Residents, Mid-Level Providers

**Time Required for Implementation:**

The instructor should expect to spend about two hours creating the model once all supplies are obtained. This time does not include shopping to collect all the supplies.

The didactic session is variable in time and will depend on the number of learners present. Using the provided materials, instructors should familiarize themselves with the pelvic examination process and review female anatomy. The learners should familiarize themselves with anatomy and the pelvic examination process using the given materials. During the trial use of this pelvic model, the training lasted anywhere from 10–30 minutes depending on learner familiarity with the pelvic exam process.

**Recommended Number of Learners per Instructor:**

The ratio can be variable. To discuss the procedure of a pelvic exam could be unlimited. When utilizing the model, the authors recommend one learner to one instructor for optimal learning.

**Topics:**

Pelvic examination, cervical examination, creative simulation models.

**Objectives:**

After utilizing this pelvic examination model, the learner will be able to:

1. Demonstrate ability to perform a pelvic examination comfortably and safely
2. Demonstrate ability to obtain a cervical swab from female patients
3. Show proficient understanding of female anatomy

### Linked objectives and methods

The goal of creating this model is to effectively teach residents, midlevel providers, and medical students how to successfully perform a pelvic examination. This training model was made as realistic as possible so the learners can translate the exam to an actual patient. Prior to preforming the exam, learners were asked by the instructor to positively identify female anatomy and landmarks and correctly describe how to operate a speculum. Once this is completed, the learner performs the speculum examination, collects the cervical swabs, and completes the exam. During the process, the instructor should provide guidance on speculum insertion and proper swabbing techniques. Once there is successful swabbing and removal of the speculum, the instructor can provide any further instruction deemed necessary, and any questions by the learner can be asked. By utilizing this format and instruction for creating the pelvic exam model, learners are afforded a hands-on realistic experience to learn how to perform a pelvic exam (objective 1) successfully and simply. This method allows learners to practice in a low stress environment and on a model as opposed to a live patient. This eliminates a lot of the stress for both the learner and the patient. The materials were selected based on what was available at a local dollar store to ensure affordability. Examples from an internet search were utilized to make a realistic model, and experience from those who contributed also assisted in creating a realistic, cost-effective model.

### Recommended pre-reading for instructor

- Bialy A, Kondagari L, Wray AA. Gynecologic Examination. StatPearls Publishing. 2023 Jan-. PubMed published 2020. https://www.ncbi.nlm.nih.gov/books/NBK534223/
- Dr James Gill. Vaginal Examination - Clinical Skills Speculum Examination Tutorial - Dr James Gill. *YouTube.* Published online September 11, 2020. Accessed April 7, 2021. https://www.youtube.com/watch?v=ys6170Q5Uqc
- Fitzgerald, G. Speculum examination. TeachMeObGyn. 2022. Accessed February 14, 2023. Available at: https://teachmeobgyn.com/history-taking-examinations/examinations/speculum/
- Hillard, P. The pelvic exam. Stanford medicine 25. YouTube. 2017. Accessed February 14, 2023. Available at: https://www.youtube.com/watch?v=AllLe3qI7uc

### Learner responsible content (LRC)

- Edelman A, Anderson J, Lai S, Braner D, Tegtmeyer K. Pelvic examination. *NEJM.* 2016. YouTube. Accessed February 14, 2023. Available at: https://www.youtube.com/watch?v=i5VxSZ9w4EY
- Hillard, P. The pelvic exam. Stanford medicine 25. YouTube. 2017. Accessed February 14,2023. Available at: https://www.youtube.com/watch?v=AllLe3qI7uc

### Implementation Methods

Emergency medicine residents, medical students, midlevel providers are introduced to the pelvic training model by the instructor who demonstrates the proper method for a speculum examination verbalizing anatomical landmarks and speculum placement. The pelvic exam model should be placed on a sturdy table and instructor(s) should have cervical collection swabs and speculums for learners to utilize. Any trained physician, resident, or midlevel provider can teach the pelvic examination. This should be hands-on learning with the home-made model as the example and the teaching tool.

The instructor should walk the learners through the exam once, using the pelvic model. Once the instructor does this, the learners should verbalize and explain the procedure back to the instructor. As the learner is inserting the speculum and collecting cervical swabs, they should be verbalizing to the instructor the landmarks and what they are visualizing. After successful completion of the exam, the instructor may then provide feedback. The learners can complete the exam as many times as they wish until they feel confident in the procedure.

### List of items required to replicate this innovation

The following materials were utilized from a local dollar store:

- Pool noodle
- Facial exfoliation pads
- 32 flat-pack cosmetic foam wedges
- Four rubber bands
- Two felt sheets
- Two wash cloths, ideally skin color but any will suffice

The following materials were used from home recycling:

- Eleven 24 oz beverage cans. In this model, empty Rockstar energy drink cans were utilized
- Empty cardboard box. The one utilized for this model measured 7″x12″x10″
- Large, wide drink straw

The following tools were utilized:

- Scissors
- Razor blade or utility knife
- Hot glue gun
- Staple gun
- Brown and red marker

### Approximate cost of items to create this innovation

Approximately $20 USD; however, this is variable depending on where the supplies are purchased.

### Detailed methods to construct this innovation

1. The first step is to prepare the cardboard box by using either scissors or the utility knife to remove the longest closure flaps on one side of the box, leaving the shorter flaps. Once this is done, lay the box vertically resting on the shorter end with the opening facing the creator.
2. Cut the pool noodle. This was used for filling in between the cans for stability, for the vaginal canal, and to hold the cervix. The noodle measured 10.5″
  - The pool noodle was cut into pieces measuring 7″ pieces to fill between cans for added stability.[Fig f1-jetem-8-3-i1]
    **Figure f1-jetem-8-3-i1:**
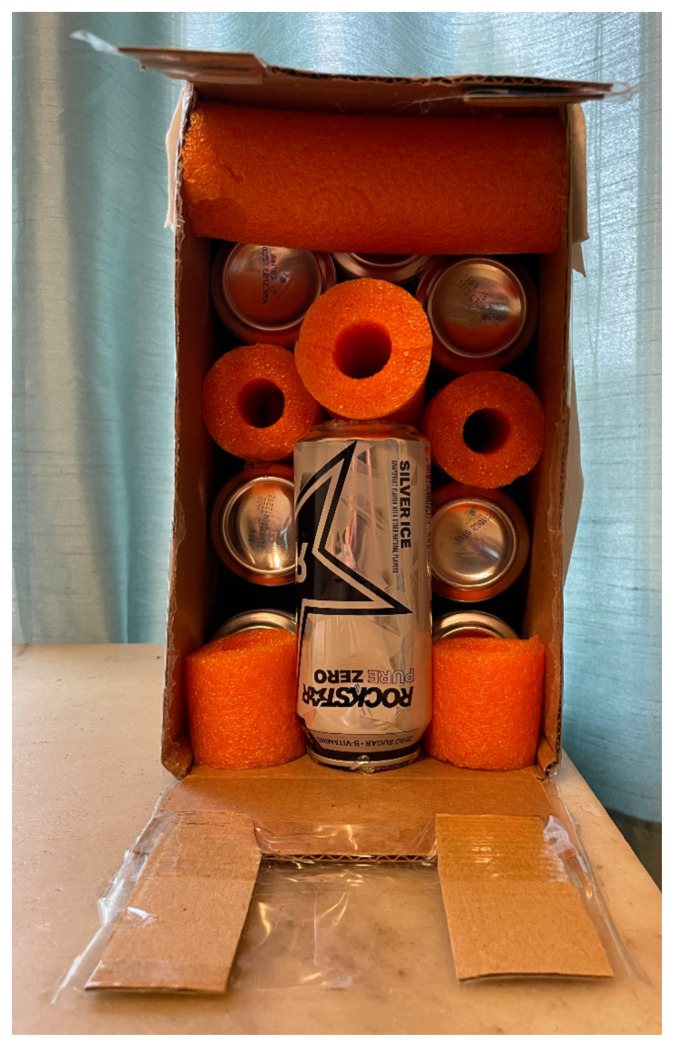
  - The pool noodle for the canal was then cut in half, length wise. The exfoliation pad was then cut to fit, mimicking a cervix. A small indentation was cut utilizing the utility knife to rest and secure the shaped exfoliation pad which was subsequently glued with the hot glue gun to the posterior aspect of the cardboard box.[Fig f2-jetem-8-3-i1]
    **Figure f2-jetem-8-3-i1:**
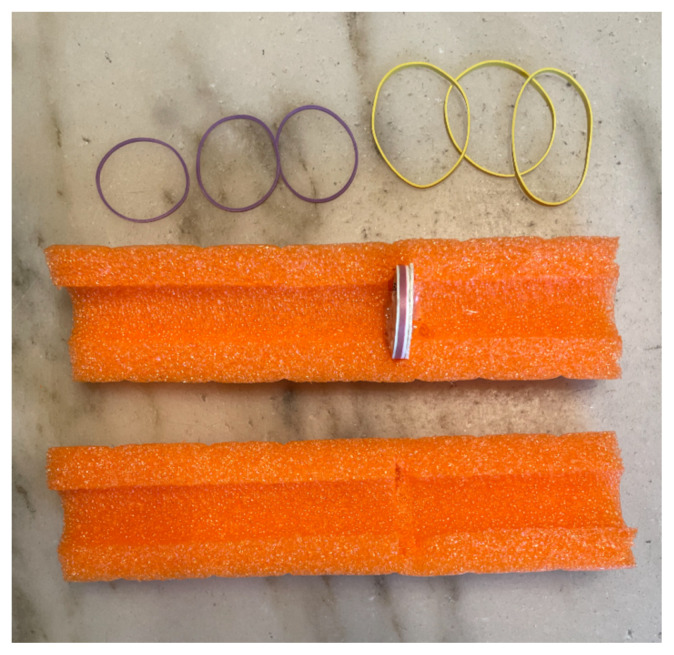
  - Four rubber bands were applied to close the canal and to allow for expansion during the speculum exam. Two cans were placed one on top of another to build a base to secure the long vaginal canal. All were inserted horizontally in the box and glued. A third can was placed vertically beneath the pool noodle canal for support. The remaining pool noodle pieces were inserted between and above to provide maximum stability.[Fig f3-jetem-8-3-i1][Fig f4-jetem-8-3-i1]
    **Figure f3-jetem-8-3-i1:**
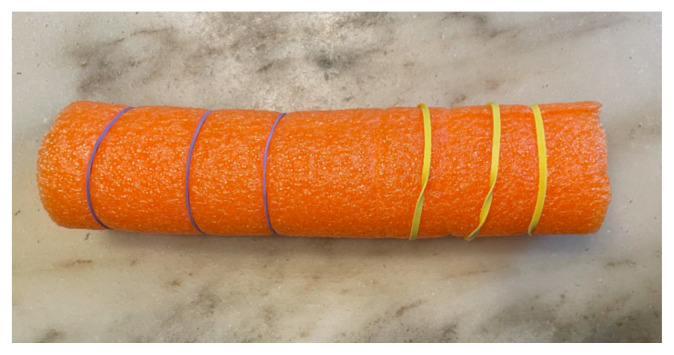

**Figure f4-jetem-8-3-i1:**
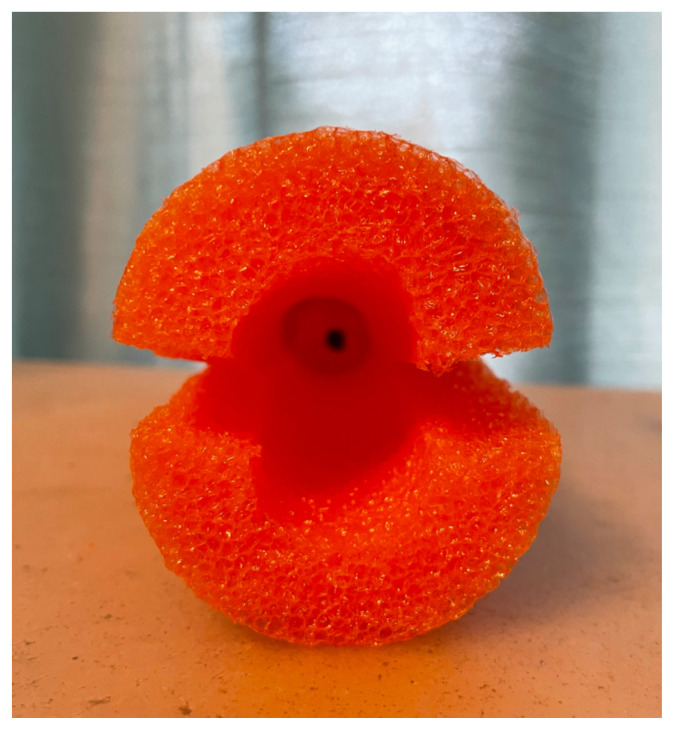
  - The two small box flaps were then closed and glued to the cans. One felt sheet was placed over the entire box face, stapled in place.[Fig f5-jetem-8-3-i1]
    **Figure f5-jetem-8-3-i1:**
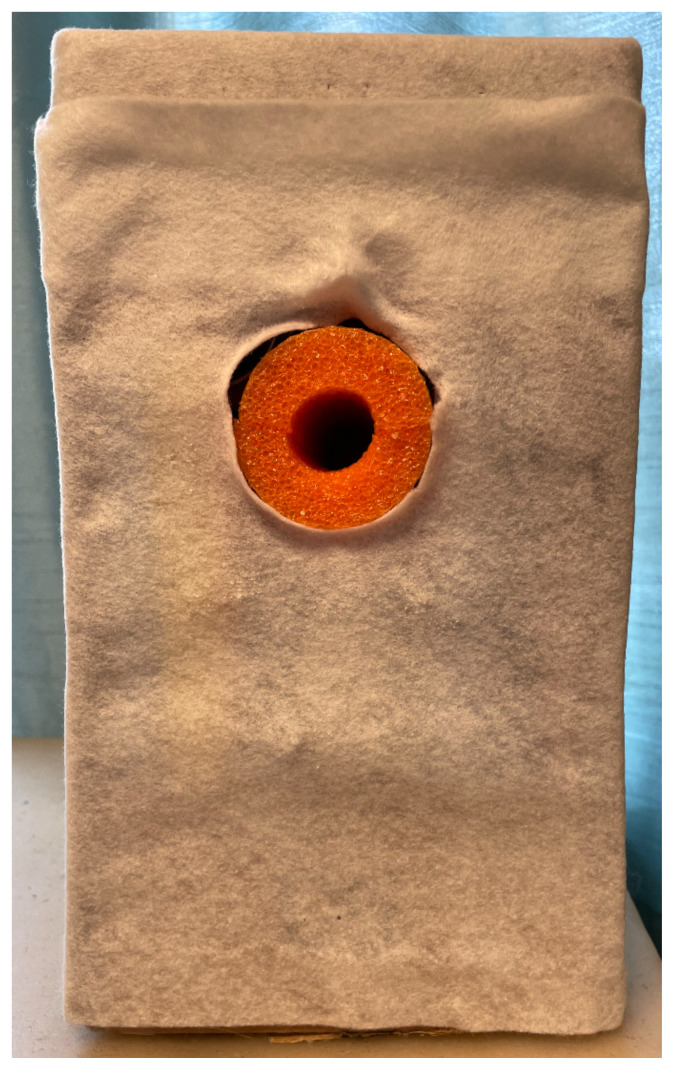
  - Cut a small vaginal opening just in front of the canal. Place the 32-count flat package of cosmetic sponges over the opening and glue securely. There should be some opening down the central line as these will mimic the labia majora. At the most apical region, cut a straw 1 inch long and glue in place to form the urethra. Secure the washcloth to the box utilizing a staple gun. We utilized a second exfoliation pad to mimic an anus which was colored with a black marker and attached with hot glue. We utilized a small cosmetic sponge for the clitoral hood and colored it pink.[Fig f6-jetem-8-3-i1]
    **Figure f6-jetem-8-3-i1:**
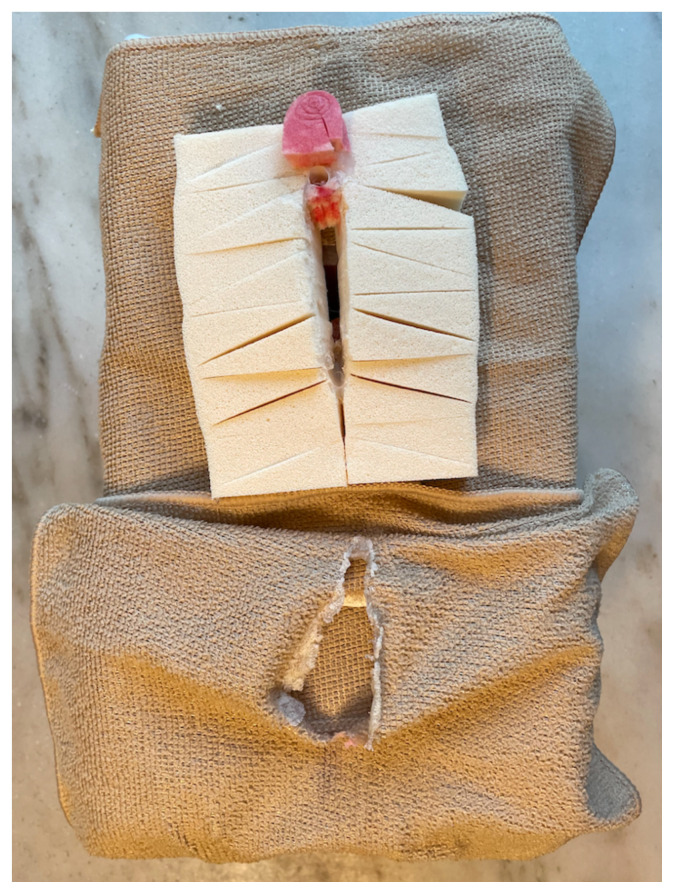
  - Secure the washcloth over the top with staples and hot glue. This cloth should also have a midline cut to provide access to the vaginal canal. Tuck the edges around the cosmetic sponges to give depth and mimic natural anatomy. Glue around the opening, securing the washcloth to the cosmetic sponges.
  - Brown and red markers were used to color around the urethra and add color to the external surface for a more realistic appearance.[Fig f7-jetem-8-3-i1]
    **Figure f7-jetem-8-3-i1:**
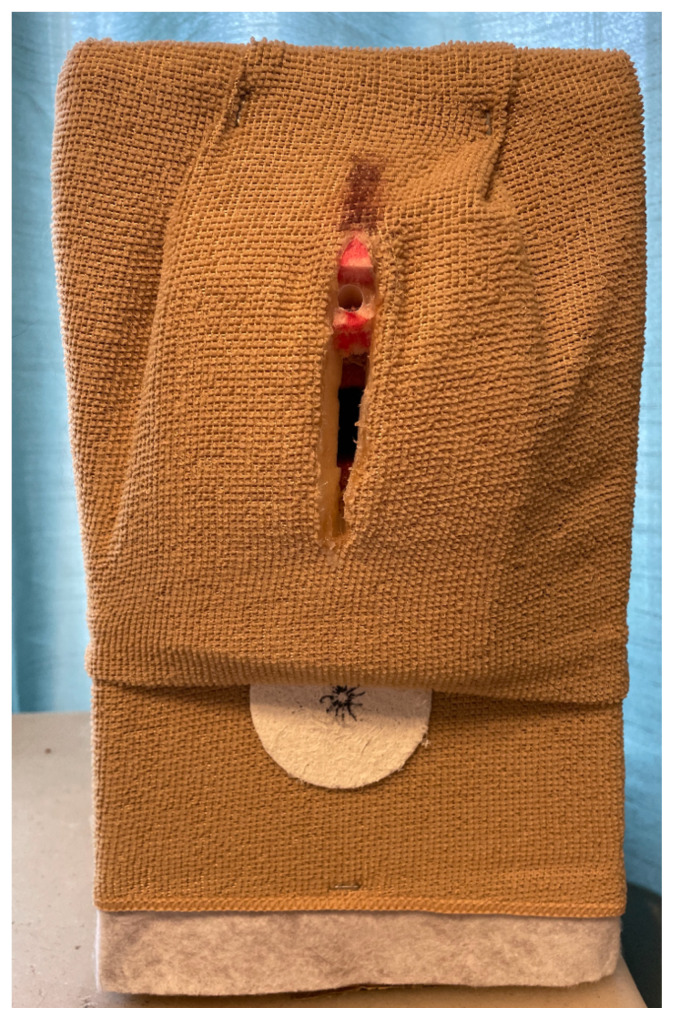

### Results and tips for successful implementation

Our model was compared side by side to a manufactured clinical female pelvic trainer by our residents during a simulation lab. The consensus found our homemade model to be comparable to the feel of a pelvic exam, as was the cervical evaluation. This model is a cost-effective way to allow for effective simulation practice for training residents in female pelvic examination techniques. Data was collected from attendings, residents, mid-level providers, and medical students across specialties of emergency medicine, family medicine, and obstetrics and gynecology presented in Table 1a, utilizing an anonymous survey from specialties at one site in Detroit, Michigan. A total of 77 individuals tested the homemade model and completed the comparison survey and tested the homemade model. Table 1b shows the distribution of training levels ranging from medical students to attendings across all three specialties.

The comparison was made between the homemade model, a commercial model (if the survey taker had experience with a commercial model within the last year), and a live patient encounter.

Among the participants, there was a variety of experience as well as frequency of performing pelvic examinations in practice. Seventy-seven per cent of the participants reported performing 15+ pelvic examinations per year, giving a high level of competency and experience with the practice of performing pelvic examinations. Comparing the commercial models to the homemade model, 54 of the 77 participants had experience with a commercial model. The goal of the survey and data collection was to determine if the homemade model felt more/less/about the same when compared to a commercial model and if utilizing the model prior to an examination on a live patient would cause the provider less stress. In the survey, 57% of the participants felt the examination was the same, and 31% indicated the homemade model felt more realistic when compared to the commercial model (see Table 2). The feedback supporting this data was the consensus that most commercial models, being made of plastic, made the advancement of the speculum more difficult when compared to the homemade model and when compared to a live patient.

When compared to a live patient, 55% of participants felt the model was less realistic, and 45% indicated the model felt the same/very similar to a pelvic exam on a live patient, as shown in Table 3.

Overall, 87% agreed that performing the examination on the homemade model was less stressful than performing an exam on a live patient and would have been a beneficial tool to improve provider and patient comfort. In total, out of the 77 participants, 99% agreed that the homemade pelvic exam model is an effective training tool when teaching pelvic examinations.

This model is best utilized in a simulation lab teaching incoming residents, mid-levels, and medical students. This model can be used effectively for all levels of learners. The model is also compact and lightweight so it can be easily transported. This model was used in our simulation lab for incoming interns and medical students and by residents across other specialties. A suggestion was also improvement in the appearance of the cervix, perhaps creating it out of a balloon for a more realistic appearance when collecting cervical swabs. Of note, the surveys were collected from one facility, which may introduce some bias in the data. Pelvic examinations will continue to be a component of clinical practice across specialties, and patient comfort is critical as is provider confidence when performing the examination. This study proved that a simple-to-manufacture, inexpensive, homemade model can be an adequate training tool. Listed below are a few of the comments obtained from the study:

- The DIY model felt closely similar to real patients. Model may be an effective training tool for residents.
- The commercial models are typically too stiff, and the plastic/rubber causes the speculum to stick/catch. The DIY model was much more realistic in both of these areas.
- This would be excellent for those learning or wishing to further develop speculum proficiency.
- Although it felt less realistic than a commercially produced model or a live patient, the DIY model is still useful for practice because it can help with getting used to inserting and opening the speculum.
- DIY pelvic model is really cool, great training concept.
- Less sticky on the speculum than the silicone official model. Cervix – maybe create a ring with material to make a bun close to speculum and create it more realistic to do swabs.
- Wonderful accuracy, very realistic, life-like.
- Very useful tool to practice doing pelvic exams, realistic
- A great tool for students/residents to practice with.
- Great effort! Very helpful to learn book skills or gain orientation.
- Overall, it seems about the same as plastic pelvic models. The main difference between models and live patients are the angle of the vagina (usually angles towards the floor instead of parallel) and the collapsibility of the vaginal side wall tissue.
- The major difference between live patients and a pelvic model is that the vagina collapses. This model was helpful for beginning learners who need practice initially placing the speculum but less so in performing the exam after initial placement/insertion.
- Will be especially useful for medical students to learn how to confidently perform pelvic exams, as patients can sometimes be hesitant to allow students to practice gaining the skill.
- Very effective tool to practice an invasive exam in a less stressful environment. Highly recommend!
- Great model to simulate live pelvic exams and be comfortable and proficient performing them.
- Effective and easy method of confirming anatomy and improving confidence for a pelvic exam.
- Very helpful as a student. Recommend for any healthcare provider in training.

## Figures and Tables

**Table 1a:**
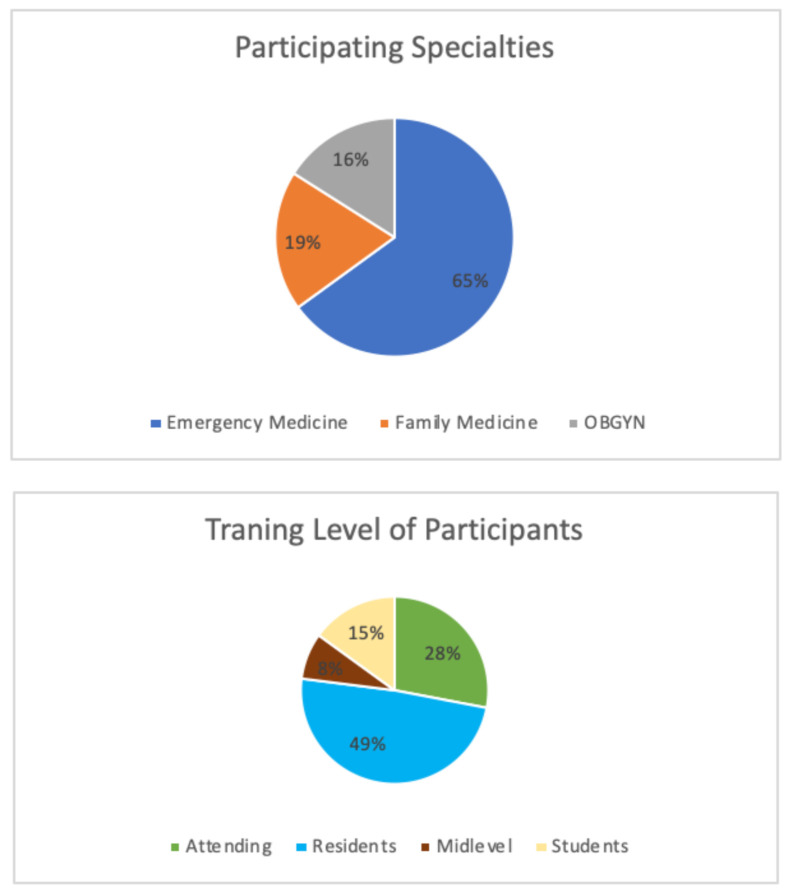


**Table 2:**
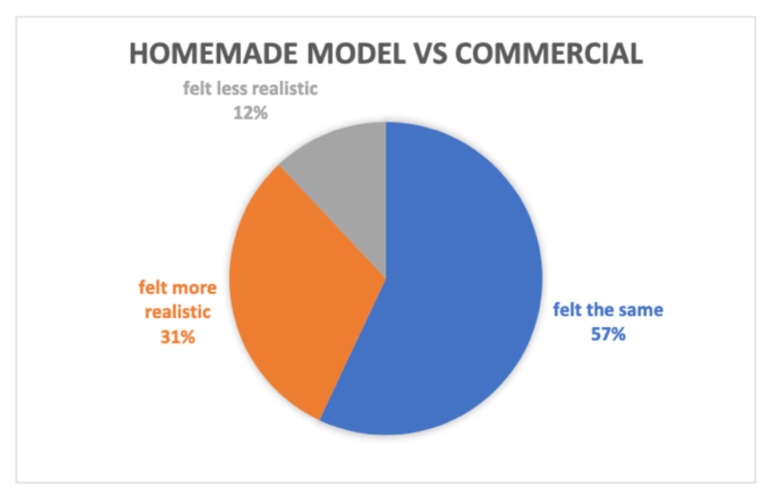


**Table 3:**